# Longitudinal Assessment of Circulating Tumor Cells and Outcome in Small Cell Lung Cancer: A Sub-Study of RASTEN—A Randomized Trial with Low Molecular Weight Heparin

**DOI:** 10.3390/cancers15123176

**Published:** 2023-06-13

**Authors:** Pär-Ola Bendahl, Mattias Belting, Emelie Gezelius

**Affiliations:** 1 Department of Clinical Sciences, Lund, Division of Oncology, Lund University, Barngatan 4, SE-221 85 Lund, Sweden; 2 Department of Hematology, Radiophysics and Oncology, Skåne University Hospital, Lasarettsgatan 23A, SE-221 85 Lund, Sweden; 3 Department of Immunology, Pathology, and Genetics, Uppsala University, Rudbecklaboratoriet, SE-751 85 Uppsala, Sweden; 4 Department of Respiratory Medicine, Lund University Hospital, Entrégatan 7, SE-221 85 Lund, Sweden

**Keywords:** small cell lung cancer, liquid biopsy, circulating tumor cells, prognostic biomarker

## Abstract

**Simple Summary:**

Small cell lung cancer (SCLC) is an aggressive lung cancer subtype associated with an overall poor prognosis but a variable response rate to chemotherapy. The measurement of circulating tumor cells (CTCs) offers a non-invasive method to monitor the disease and may provide prognostic information as potential guidance to clinicians in the management of SCLC. However, the value of CTCs during and after chemotherapy appears inconclusive. Here, we show that the detection of CTCs at baseline correlates to overall survival in SCLC, and that persistently detectable CTCs after completion of treatment adds further prognostic value. This suggests that repetitive analysis of CTCs during and after the course of treatment may have a role in the management of SCLC, warranting further studies.

**Abstract:**

Circulating tumor cells (CTCs) may provide a liquid biopsy approach to disease monitoring in small cell lung cancer (SCLC), a particularly aggressive tumor subtype. Yet, the prognostic role of CTCs during and after treatment in relation to baseline remains ill-defined. Here, we assessed the value of longitudinal CTC analysis and the potential of low-molecular-weight heparin (LMWH) to reduce CTC abundance in SCLC patients from a randomized trial (RASTEN). Blood samples were collected at baseline, before chemotherapy Cycle 3, and at 2-month follow-up from 42 patients in total, and CTCs were quantified using the FDA-approved CellSearch system. We found a gradual decline in CTC count during and after treatment, independently of the addition of LMWH to standard therapy. Detectable CTCs at baseline correlated significantly to reduced survival compared to undetectable CTCs (unadjusted hazard ratio (HR) of 2.75 (95% CI 1.05–7.20; *p* = 0.040)). Furthermore, a persistent CTC count at 2-month follow-up was associated with a HR of 4.22 (95% CI 1.20–14.91; *p* = 0.025). Our findings indicate that persistently detectable CTCs during and after completion of therapy offer further prognostic information in addition to baseline CTC, suggesting a role for CTC in the individualized management of SCLC.

## 1. Introduction

Small cell lung cancer (SCLC), accounting for 13% of all lung cancer cases [1], is characterized by early metastatic spread and a particularly poor prognosis, with 2-year survival rates of ~5% in patients with extensive disease (ED) at diagnosis [1,2]. SCLC is a dynamic disease, typically associated with high response rates to initial therapy and often dramatic symptomatic relief. However, a majority of patients experience disease relapse, with limited therapeutic options and poor response to subsequent treatment lines [3,4]. The mechanisms behind the emerging resistance to platinum-based chemotherapy are not yet fully understood, and the early identification of patients with platinum-resistant or -refractory disease remains a challenge.

Diagnosis and monitoring of SCLC mainly relies on radiographic assessment, as repeated tissue sampling through tumor biopsies is not feasible. Hence, there is a need for tools that allow more dynamic and quantitative evaluation of tumor aggressiveness and treatment response. Here, liquid biopsy, based on the measurement of circulating tumor cells (CTCs), offers a non-invasive method to monitor disease extent and provide prognostic information as potential guidance for clinicians in the management of SCLC. In addition, the molecular characterization of CTCs is an evolving field that may provide further insight into the biology of SCLC [5].

Compared to non-small cell lung cancer (NSCLC), CTCs are often abundant in SCLC [6,7], which may reflect the propensity for rapid proliferation and dissemination. Several studies have investigated the prognostic value of CTCs in SCLC [8,9,10,11,12,13,14,15], comprehensively reviewed by De Luca et al. [16]. It is generally concluded that the number of CTCs at the time of diagnosis indicates an unfavorable prognosis. This is supported by a meta-analysis of seven studies including 440 SCLC patients reporting an association between reduced survival and the presence of CTCs [17]. However, the role of subsequent CTC measurement appears inconclusive, which may partly be explained by a great heterogeneity in terms of CTC thresholds and time points for repeated measurements. An association between CTC number change and outcome has been demonstrated by some studies [11,15], and Normanno et al. [18] reported a reduction of 89% or more to be prognostic. Still, others have not found any significant correlation between the change in CTC count after the start of chemotherapy and survival [19,20].

The aim of the present study was to explore the prognostic value of CTC presence in SCLC, dynamically assessed in longitudinal samples from a patient cohort originating from the RASTEN phase III trial, a randomized controlled study that assessed the survival benefit of the addition of low-molecular-weight heparin (LMWH) to standard chemotherapy [21]. A major biological rationale of RASTEN and other trials that explore the anti-tumoral and survival effects of LMWH was based on previous experimental studies indicating reduced levels of circulating, metastatic cells by LMWH [22]. Hence, we hypothesized that patients receiving additional LMWH would demonstrate a greater reduction in CTC numbers during treatment compared to patients treated with chemotherapy only.

## 2. Materials and Methods

### 2.1. RASTEN Patient Cohort

The patients included in this biomarker cohort were originally enrolled in a randomized phase III trial investigating the effects of LMWH on survival in SCLC (RASTEN; ClinicalTrials.gov: NCT00717938) [21]. Standard therapy within the trial included a platinum compound combined with a topoisomerase inhibitor, and radiotherapy was administered according to local protocol guidelines. In addition, patients randomized to the intervention arm received the LMWH enoxaparin for the duration of the chemotherapy regimen. The trial was conducted in agreement with the Declaration of Helsinki and with approval from the Regional Ethics Committee at Lund University, Sweden. Written, informed consent was obtained from all participants, including specific consent for the collection and analysis of blood samples. RASTEN is a multi-center trial, but for methodological reasons related to CTC analysis, patients in this substudy were all included at the Skåne University Hospital, Lund. The patients were consecutively included at this site, minimizing the risk of other selection bias.

### 2.2. Sampling and Analysis of Circulating Tumor Cells

Counting of CTCs in the blood circulation was performed at three different time points: at baseline (prior to chemotherapy), before Cycle 3, and at follow-up 2 months after completion of chemotherapy, using the Food and Drug Administration (FDA)-approved CellSearch^®^ method (Veridex, Raritan, NJ, USA). Peripheral blood was collected in CellSave preservative tubes (7.5 mL) (Veridex). The first 3–5 mL of each blood withdrawal were discarded before collection of samples for CTC analysis to avoid contamination with skin epithelial cells. Samples were maintained at room temperature and further processed within 48 h. The precision, accuracy, and reproducibility of CTC measurements using the CellSearch^®^ system have been described previously [6]. In brief, ferro-fluid particles conjugated with antibodies to epithelial cell adhesion molecule (EpCAM) were used to capture cells expressing the antigen. Unbound cells were excluded, and the enriched sample was fluorescently labelled for nuclei (DAPI), cytokeratins (CKs 8, 18, and 19), and CD45. Cells with a size of >4 μm presenting the phenotype DAPI+/CK+/CD45- were defined as CTCs. All CTC evaluations were performed at the Department of Oncology, Clinical Sciences, Lund University, Sweden, by two accredited and independent scorers.

### 2.3. Clinical Outcome

The primary outcome, overall survival (OS), was defined as the date of randomization to the date of death from any cause. For patients not reported dead, information regarding vital status was confirmed from the study center before data collection cut-off on 4 April 2017.

### 2.4. Statistical Analysis

Data were analyzed using the statistics programs IBM SPSS Statistics, Version 27.0. Armonk, NY, USA and STATA 17, StataCorp LLC, TX, USA. Non-parametric methods were used for comparisons of CTC levels in different subgroups (exact Mann–Whitney test) and over time (exact Wilcoxon matched-pairs signed-rank test). The user-contributed Stata routine spagplot was used to draw spaghetti plots of the CTC count over the three sampling time points: at baseline, at the start of Cycle 3 and at the 2-month follow-up. For improved visualization, a small random number drawn from a uniform distribution over the interval [−0.1, 0.1] was added to each observed CTC count to separate identical counts and lines. The threshold of ≥1 was used to define CTC positivity. Patients were further categorized according to change in CTC detection from baseline to Cycle 3 or 2-month follow-up, respectively, and defined as +/+ if CTC count was persistently positive, +/− if CTCs were detectable at baseline but not at the subsequent time point, and −/− if CTCs remained undetectable. The Kaplan–Meier method was used to estimate survival and the log rank test was utilized to assess the evidence for difference in survival between patient groups. Cox regression was used to estimate hazard ratios (HRs). Assumptions of proportional hazards were checked graphically. Multivariable Cox models were used to calculate HRs adjusted for age (linear), gender, disease stage and performance status (WHO 0–1 vs. 2–3). Landmark analysis was used when including CTC values at Cycle 3 or 2-month follow-up in survival analyses [23].

## 3. Results

### 3.1. Patient Characteristics

Baseline samples for CTC analysis were available in 46 patients, of which four were excluded due to non-evaluable assay results. In total, 42 patients were included in the present cohort, of which 21 (50%) had limited disease (LD) and the other 50% had extensive disease (ED). Patient demographics, presented in Supplementary Table S1, show similar characteristics for the two study arms in this substudy of the randomized trial. All patients initiated at least one cycle of chemotherapy, and the 20 patients allocated to the LMWH arm received additional enoxaparin as per clinical trial protocol. The median length of follow-up was 23 months (range 13–81) for patients who were still alive, and median overall survival was 12.5 months for the whole cohort.

### 3.2. Circulating Tumor Cell Distribution at Baseline

CTCs were detected in 74% of cases (31/42) with a median CTC count of 11 (range 0–4434; Table 1). The CTC count was higher in patients with ED compared to those with LD (*p* = 0.002). In LD, CTCs were detected in 62% of cases compared to 86% in ED patients, with median counts of 3 vs. 49 in LD and ED, respectively (Table 1). The data do not support any difference in CTC distribution by gender (male vs. female, median CTC count 15 vs. 7; *p* = 0.475) or performance status (0–1 vs. 2–3, median CTC count 7 vs. 49; *p* = 0.171).

### 3.3. Circulating Tumor Cells during and after Treatment

**Figure 1:**
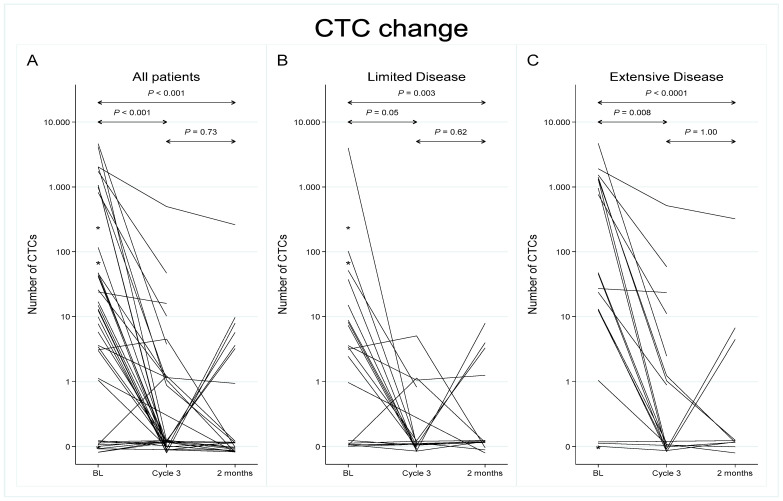
Longitudinal analysis of circulating tumor cells from baseline to chemotherapy Cycle 3 and at a 2-month follow-up. All patients are represented in panel (**A**) (*N* = 42), while panels (**B**,**C**) display patients with limited disease (*N* = 21) and extensive disease (*N* = 21), respectively. Note: The *y*-axis scale is logarithmic, base 10. CTC counts of zero are plotted at y = 0.1 on this scale, a point which therefore has been labelled 0. * Patients where baseline samples only were available for analysis (*N* = 3).

A significant reduction in CTC enumeration was observed during chemotherapy (prior to start of Cycle 3) and at 2-month follow-up compared to baseline levels, both in LD and ED (Table 1 and Figure 1). During treatment, detectable CTCs were found in 33% of the 36 cases available for analysis (median = 0; range 0–452), and the difference in distribution across disease stage diminished. Eleven patients had persistently detectable CTC counts at baseline and at Cycle 3 (+/+), whereas 17 patients with baseline CTC ≥ 1 had undetectable levels during chemotherapy (+/−). The CTC count remained negative from baseline to Cycle 3 in seven cases (−/−). Only one patient with undetectable CTCs at baseline had a positive CTC count at chemotherapy Cycle 3, which reverted back to zero at 2-month follow-up (−/+/−) and was excluded from subsequent survival analysis. At follow-up 2 months after completion of treatment, CTCs were present in 7 (29%) out of 24 evaluable cases. CTC numbers remained low (median = 0; range 0–280), although five patients reverted to a positive CTC count (+/−/+) (Figure 1 and Table 1).

### 3.4. Effect of LMWH on CTC Enumeration

In total, 18 of the 22 patients enrolled in the control arm had evaluable CTC counts at chemotherapy Cycle 3. Median CTC numbers decreased from 14 at baseline to 0 during chemotherapy (median reductions of 14 CTCs). Similarly, in the LMWH arm, CTC numbers were reduced from 12.5 at baseline to 0 at Cycle 3 (median reductions in 10 CTCs) in the 18 patients with blood samples available at both time points (Supplementary Figure S1). In line with the negative outcome of the RASTEN trial [21], there was no difference in change in CTC count between baseline and Cycle 3 when comparing patients in the control arm and in the intervention arm, receiving LMWH (*p* = 0.969).

### 3.5. Prognostic Significance of CTCs at Baseline

**Figure 2:**
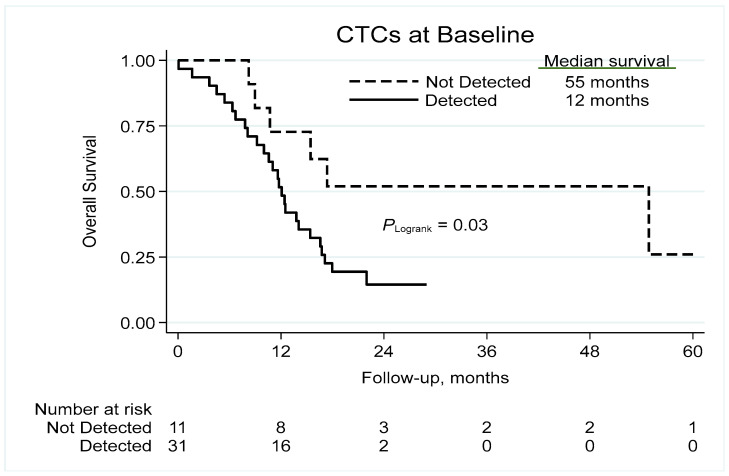
Kaplan–Meier analysis of overall survival based on the detection of CTCs. CTC = circulating tumor cell.

The presence of CTCs at baseline correlated significantly to reduced survival, with median overall survival of 12 and 55 months in patients with and without detectable CTCs, respectively, corresponding to 2-year survival rates of 15% and 52% (Figure 2), and a univariable HR of 2.75 (95% confidence interval (CI) 1.05–7.20; *p* = 0.040; Table 2). Adjustment for stage, age (linear), gender and performance status (0–1 vs. 2–3) resulted in a HR of 2.11 (95% CI 0.78–5.70; *p* = 0.140).

Subgroup analysis by disease extent revealed numerically strong but non-significant differences in survival by presence/absence of CTCs at baseline (Supplementary Figure S2); in LD, the unadjusted HR was 2.70 (95% CI 0.73–9.92; *p* = 0.136) and in ED, the unadjusted HR was 2.09 (95% CI 0.47–9.30; *p* = 0.333).

### 3.6. Change in CTC Count and Clinical Outcome

**Figure 3:**
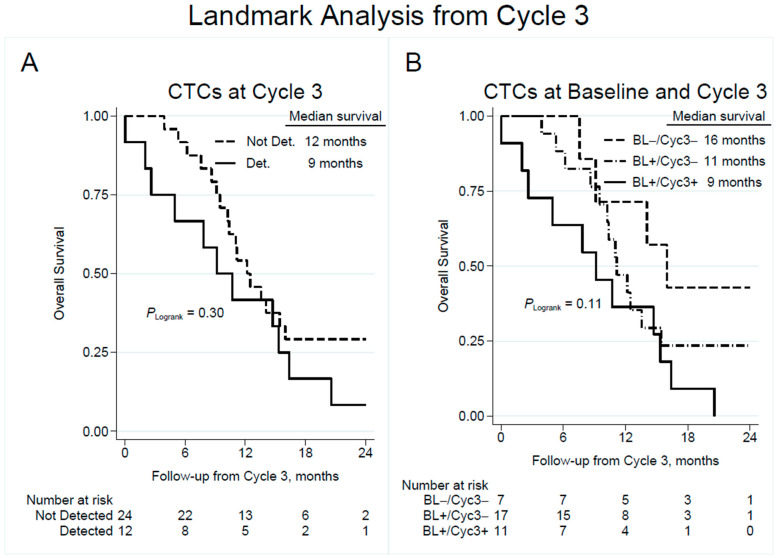
Overall survival by detection of CTCs at blood collection prior to chemotherapy Cycle 3 (**A**), with subgroup analysis showing the effect of change in CTC detection from baseline to Cycle 3 (**B**). Note that time point zero corresponds to the time of CTC sampling at Cycle 3. CTC = circulating tumor cell; Not Det. = not detected; Det. = detected; BL = baseline; Cyc3 = chemotherapy Cycle 3.

The estimated survival at 6 months from start of Cycle 3 was 92% in patients with undetectable CTCs and 67% in patients with detectable CTCs at Cycle 3 (Figure 3A). At 12 months from CTC sampling at Cycle 3, the survival was 54% and 42% in patients without and with CTCs, respectively, implying that the prognostic value of CTC detection during treatment declined over time.

Landmark analysis of overall survival from Cycle 3 based on change in CTC detection at Cycle 3 vs. baseline (Figure 3B) showed an estimated survival at 6 months of 100%, 88%, and 64% in the CTC (−/−), (+/−), and (+/+) patient categories, respectively. Corresponding results for median OS were 16 months, 11 months and 9 months, suggesting that patients with a persistent negative CTC count had the best prognosis, while patients that shifted from a positive to negative CTC count were intermediate, and patients with a constantly positive CTC count had a worse outcome. Unadjusted Cox regression analysis revealed that persistently detectable CTCs at Cycle 3 were associated with an unadjusted HR of 3.15 (95% CI 1.00–9.98; *p* = 0.051) compared to patients that remained negative in CTC count (Table 2).

**Figure 4:**
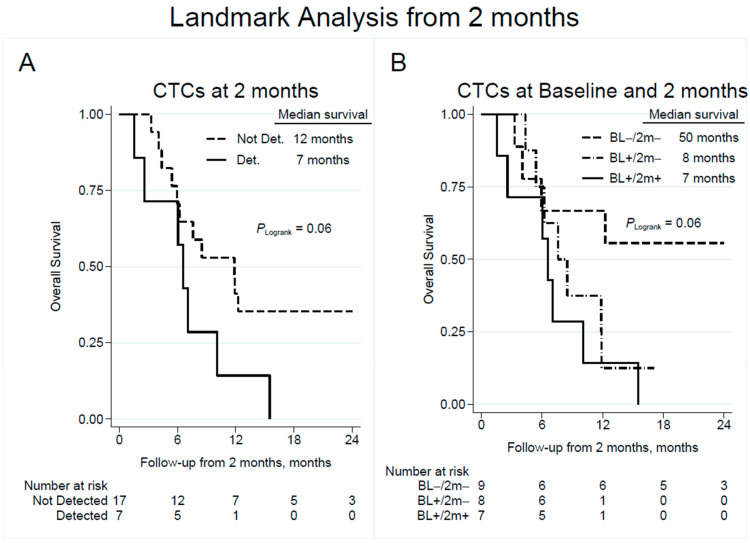
Overall survival by detection of CTCs at follow-up 2 months after completion of chemotherapy (**A**) with subgroup analysis showing the effect of change in CTC detection from baseline to follow-up (**B**). Note that time point zero corresponds to the time of CTC sampling at a 2-month follow-up. CTC = circulating tumor cell; Not Det. = not detected; Det. = detected; BL = baseline; 2 m = 2-month follow-up.

A corresponding landmark analysis based on CTC count at a 2-month follow-up yielded a median OS of 12 and 7 months in patients with undetectable and detectable CTCs, respectively, with an unadjusted HR of 2.43 (95% CI 0.93–6.38; *p* = 0.071; Figure 4A and Table 2). Compared to patients with persistently negative CTC counts, survival from a 2-month follow-up was significantly reduced in patients with continuously detectable CTCs (unadjusted HR 4.22; 95% CI 1.20–14.91; *p* = 0.025; Figure 4B and Table 2). Multivariable Cox regression models did not reveal any associations between CTC positivity and survival at the different time points (Supplementary Table S2).

## 4. Discussion

In this study, we show that persistently detectable CTCs in follow-up samples correlate to an unfavorable prognosis, supporting the value of longitudinal monitoring of CTCs in SCLC. Previous studies have shown that CTCs are frequently present in SCLC and are generally perceived to reflect a high metastatic potential [10]. Consistently, our results show significantly higher baseline CTC counts in patients with extensive compared to limited disease. In addition, we found associations between the presence of CTCs at baseline and reduced survival, in line with previous studies [8,9,10,13]. Although the study is exploratory in nature and needs confirmation by further investigations, our findings lend support to the notion that in patients with previously detected baseline CTCs, the assessment of CTC enumeration after completion of primary treatment may serve as a marker of early disease progression. Survival analysis of CTC count at the 2-month follow-up alone or a change in CTC count from baseline to chemotherapy Cycle 3 did not reach statistical significance, which may in part be explained by the limited sample size.

The role of CTCs as a potential liquid biopsy has been extensively studied in SCLC, and several advantages have been identified compared to tissue biopsies, including the non-invasive nature of the method and repeatability over time. Still, CTCs have not yet been implemented in clinical practice, as there are analytical issues to overcome. Despite efforts to define the optimal cut-off for CTC enumeration as a prognostic tool, no consensus has been reached, which limits its clinical utility [16]. Previous studies have displayed a heterogeneity in terms of analytical and statistical methods, with proposed thresholds ranging from 2 to 50 CTCs per 7.5 mL of whole blood. The present study was not powered to test for optimal cut-offs. Instead, we pragmatically chose the binary categorization of CTCs being detected or not detected. The great variation in thresholds makes direct comparisons between studies difficult. Yet, in line with our findings, Naito et al. [15] identified patients with a good, intermediate and poor prognosis, respectively, based on the change in CTC count from baseline to sampling 3 weeks after completion of treatment, using ≥ 8 as a cut-off. Similarly, with a threshold of ≥50 CTCs, Hou et al. [11] reported strong correlations between overall survival and change from baseline CTC count to sampling after the first chemotherapy cycle. Another limitation concerning the impact of repeated CTC quantifications is the uncertainty regarding the optimal timing of follow-up samples, giving rise to a spectrum of different sampling points, as detailed above [16]. This brings limitations to what conclusions can be drawn and how longitudinal assessment can be applied. The dynamics of CTC enumeration during and after treatment are intriguing. The CTC count has been found to correlate to tumor burden, as assessed radiographically, and several studies have shown significant reductions after start of chemotherapy [11,14,19]. Still, a decline in CTC numbers has not been found to associate with objective response [10,15].

The RASTEN cohort also allowed the direct assessment of how anticoagulant LMWH may affect CTC levels. Cancer is associated with a hypercoagulable state and an increased risk of venous thromboembolism (VTE) [24]. Preclinical evidence has suggested that several of the key coagulation factors are not only involved in the development of VTE, but also contribute to tumorigenic processes such as metastasis and angiogenesis [25]. Heparin and heparin derivatives (LMWH) have exhibited tumor-inhibiting effects in vitro [26,27,28], and early clinical trials have demonstrated improved survival with the addition of LMWH and other anticoagulants, specifically in patients with SCLC [29,30,31]. However, in line with other contemporary studies [32,33], the RASTEN trial did not demonstrate any survival benefit with the addition of LMWH. Consistently, the present study did not demonstrate any differences in CTC reduction during treatment between patients receiving chemotherapy alone or in combination with LMWH. These data indicate that CTCs are not targeted by LMWH, lending further support to the conclusions that LMWH cannot be recommended as an adjuvant tumor-inhibiting agent in SCLC.

Besides the prognostic value of CTC count, the molecular characterization of CTCs is a growing field that may provide important information about tumor biology. As such, CTCs hold potential as surrogate markers of primary tumor tissue and may display distinct profiles with differential therapeutic responses. For example, CTCs have been found to express Schlafen 11 (SLFN11), a DNA/RNA helicase that predicts response to several DNA-damaging agents [34,35]. The Delta-like ligand-3 (DLL3), an atypical Notch ligand, which is highly expressed by neuroendocrine cancer cells, is currently being explored as a therapeutic target in SCLC [36]. Here, the evaluation of DLL3 expression on CTCs may guide future DLL3-targeting therapies [37]. Moreover, the establishment of CTC-derived xenograft (CDX) models enables extensive genetic and epigenetic studies of SCLC, and, importantly, it has been found to mirror the therapeutic response of the donor patient [38,39]. However, despite these efforts, the predictive value of CTC profiling for treatment selection remains to be further explored in order to reach clinical use.

The quantification and characterization of CTCs represents one of many facets of liquid biopsies. Other methods include the evaluation of circulating tumor DNA (ctDNA) and extracellular vesicles (EV). EVs are membrane-derived microparticles that can be shed from any cell, especially in conditions associated with cellular stress such as cancer [40,41]. EVs are involved in intercellular communication and carry surface proteins from the cell of origin. Thus, EVs provide possibilities to dynamically study tumor proteomics, metabolomics, transcriptomics and genomics [40,42]. The analysis of ctDNA is already implemented as a clinical tool for the detection of oncogenic driver mutations in NSCLC, both in the diagnostic setting and in search for acquired resistance mutations [43]. In contrast to NSCLC, to date, there are no known targetable genetic alterations in SCLC, hence ctDNA does not yet have a role in the care of SCLC patients. However, Chemi et al. [38] have recently demonstrated that distinct methylation profiles can be detected in circulating, cell-free DNA from SCLC patients, with correlations to disease stage, survival, and molecular SCLC subtypes. Together with the emerging advances in the field, liquid biopsies extending beyond CTC enumeration may be an integrated part of personalized medicine in SCLC in the future.

## 5. Conclusions

To conclude, our findings suggest that persistently detectable CTCs during and after completion of therapy offer further prognostic value in addition to baseline CTC count. A consensus regarding optimal thresholds and follow-up samples is highly warranted in order to ultimately determine the role of CTCs in the individualized management of SCLC patients.

## Figures and Tables

**Table 1:** Longitudinal distribution of CTCs in all patients and by disease stage.

	Number of Samples with Detectable CTCs (%)	Median CTC Count	Range
Baseline			
All patients, *N* = 42	31 (74)	11	0–4434
LD, *N* = 21	13 (62)	3	0–3977
ED, *N* = 21	18 (86)	49	0–4434 *
At chemotherapy Cycle 3			
All patients, *N* = 36	12 (33)	0	0–452
LD, *N* = 17	4 (24)	0	0–5
ED, *N* = 19	8 (42)	0	0–452
At 2-month follow-up			
All patients, *N* = 24	7 (29)	0	0–280
LD, *N* = 14	4 (29)	0	0–8
ED, *N* = 10	3 (30)	0	0–280

CTC = circulating tumor cell; LD = limited disease; ED = extensive disease. Difference in CTC distribution comparing limited to extensive disease using exact Mann–Whitney test, noted as * *p* < 0.05

**Table 2:** Unadjusted effects of CTC detection on overall survival at each time-point and relative to baseline detection.

	HR (95% CI)	*p*-Value
Baseline		
CTC = 0	1 (ref.)	
CTC ≥ 1	2.75 (1.05–7.20)	0.040
At chemotherapy Cycle 3		
CTC = 0	1 (ref.)	
CTC ≥ 1	1.49 (0.70–3.17)	0.301
At 2-month follow-up		
CTC = 0	1 (ref.)	
CTC ≥ 1	2.43 (0.93–6.38)	0.071
Change from baseline to Cycle 3		
−/−	1 (ref.)	
+/−	1.88 (0.61–5.84)	0.272
+/+	3.15 (1.00–9.98)	0.051
Change from baseline to 2-month follow-up		
−/−	1 (ref.)	
+/−	2.79 (0.79–9.83)	0.111
+/+	4.22 (1.20–14.91)	0.025

CTC = circulating tumor cell; HR = hazard ratio; CI = confidence interval. (−/−) = undetectable CTCs at baseline and subsequent sampling. (+/−) = detectable CTCs at baseline, undetectable at subsequent sampling. (+/+) = detectable CTCs at baseline and subsequent sampling.

## Data Availability

The data presented in this study are available from the corresponding author on reasonable request.

## Supplementary Materials

The following supporting information can be downloaded at: https://www.mdpi.com/article/10.3390/cancers15123176/s1, Table S1: Study population baseline characteristics by study arm, Table S2: Adjusted effects of CTC detection on overall survival at each time-point, Figure S1: Longitudinal analysis of circulating tumor cells from baseline to chemotherapy Cycle 3 and at 2-month follow-up by study arm, Figure S2: Kaplan–Meier analysis of overall survival by detection of baseline CTCs and by stage.
