# Supplementary material for: Longitudinal Assessment of Circulating Tumor Cells and Outcome in Small Cell Lung Cancer: A Sub-Study of RASTEN—A Randomized Trial with Low Molecular Weight Heparin
Source: Cancers (Basel). 2023 Jun 13;15(12):3176. doi: 10.3390/cancers15123176 (PMC10295929; doi:10.3390/cancers15123176)
Supplement: Supplementary file 1 [file cancers-15-03176-s001.zip › cancers-2312266-supplementary.pdf]

Supplementary Figure S1: Longitudinal analysis of circulating tumor cells from baseline to chemotherapy cycle 3 and at 2-month follow-up, by study arm.

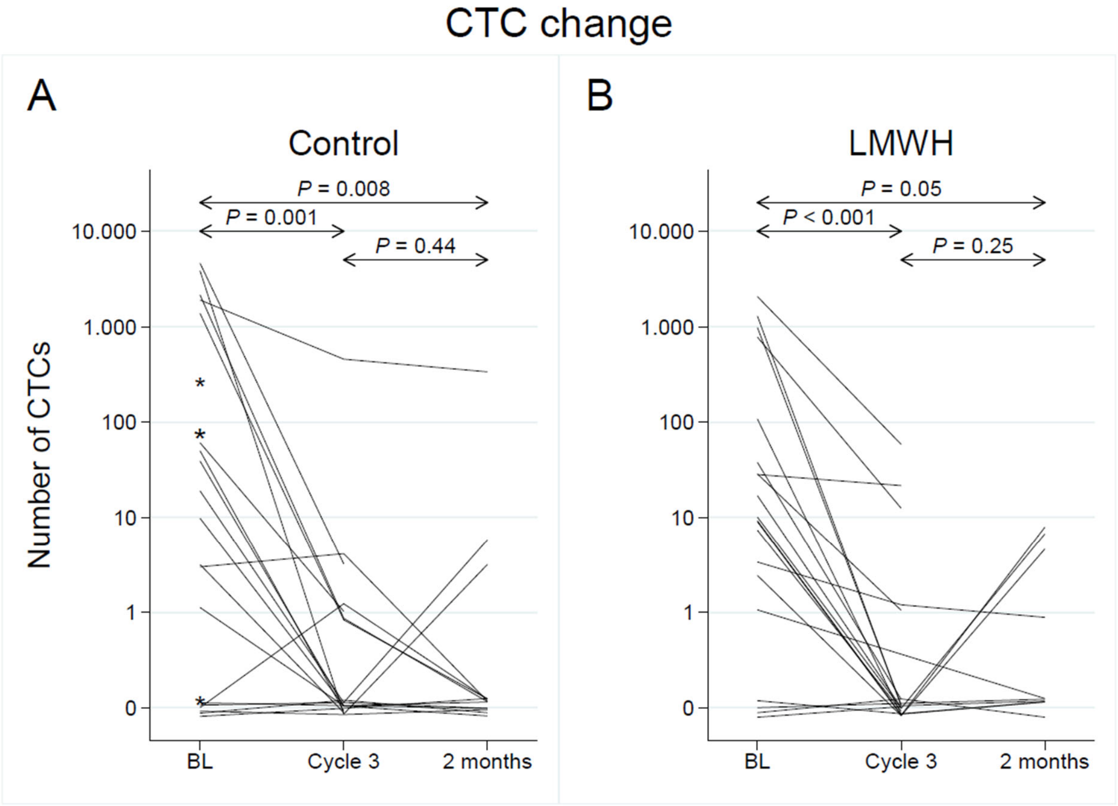

Panel A represents patients in the control arm (*N*=22), while panel B displays patients in the LMWH arm (*N*=20). Note: The y-axis scale is logarithmic, base 10. CTC counts of zero are plotted at y=0.1 on this scale, a point which therefore has been labelled 0. CTC = circulating tumor cell. *Patients where baseline samples only were available for analysis (*N*=3).

Supplementary Figure S2: Kaplan-Meier analysis of overall survival by detection of baseline CTCs and by stage; limited (A) and extensive (B) disease.

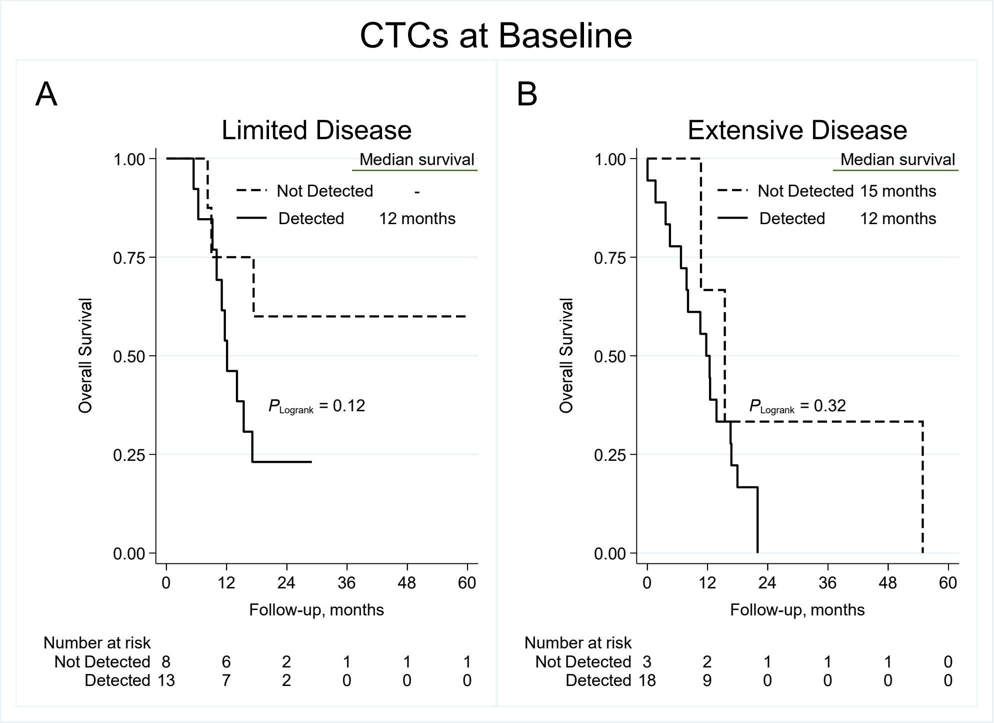

CTC = circulating tumor cell.

Supplementary Table S1. Study population baseline characteristics, by study arm.

	**LMWH** ***N*=20**	**Control** ***N*=22**
**Age, years**		
Mean ± SD	67 ± 7.6	64 ± 5.8
**Gender, *N* (%)**		
Female	12 (60)	11 (50)
Male	8 (40)	11 (50)
**Performance status, *N* (%)**		
0-1	14 (70)	17 (77)
2-3	6 (30)	5 (23)
**Disease stage, *N* (%)**		
Limited disease	11 (55)	10 (45)
Extensive disease	9 (45)	12 (55)
**Number of chemotherapy cycles, *N* (%)**		
< 4	1 (5)	1 (5)
≥ 4	19 (95)	21 (95)
**Additional chemotherapy, *N* (%)**		
1 subsequent line	7 (35)	7 (32)
≥2 subsequent lines	1 (5)	2 (9)
No additional chemotherapy	12 (60)	13 (59)
**Radiotherapy, *N* (%)***		
Prophylactic cranial	13 (72)	15 (71)
Thoracic	12 (67)	13 (62)
Metastatic lesion	4 (22)	5 (24)
No radiotherapy	2 (11)	3 (14)
Missing	2	1

LMWH = Low-molecular-weight heparin; SD = Standard deviation.

* Patients may have received radiotherapy towards more than one site.

Supplementary Table S2. Adjusted effects of CTC detection on overall survival at each time-point.

	HR (95% CI)	*P*-value
**Baseline**		
CTC = 0	1 (ref.)	
CTC ≥ 1	2.11 (0.78-5.70)	0.140
**At chemotherapy cycle 3**		
CTC = 0	1 (ref.)	
CTC ≥ 1	2.26 (0.92-5.57)	0.076
**At 2-month follow-up**		
CTC = 0	1 (ref.)	
CTC ≥ 1	2.31 (0.79-6.74)	0.126

Adjusted for disease stage, age (linear), gender and performance status (0-1 *vs.* 2-3).
CTC = circulating tumor cell; HR = hazard ratio; CI = confidence interval.
